# Performance of Artificial Intelligence Models Designed for Automated Estimation of Age Using Dento-Maxillofacial Radiographs—A Systematic Review

**DOI:** 10.3390/diagnostics14111079

**Published:** 2024-05-22

**Authors:** Sanjeev B. Khanagar, Farraj Albalawi, Aram Alshehri, Mohammed Awawdeh, Kiran Iyer, Barrak Alsomaie, Ali Aldhebaib, Oinam Gokulchandra Singh, Abdulmohsen Alfadley

**Affiliations:** 1 Preventive Dental Science Department, College of Dentistry, King Saud Bin Abdulaziz University for Health Sciences, Riyadh 11426, Saudi Arabia; 2 King Abdullah International Medical Research Center, Ministry of National Guard Health Affairs, Riyadh 11481, Saudi Arabia; 3 Restorative and Prosthetic Dental Sciences Department, College of Dentistry, King Saud bin Abdulaziz University for Health Sciences, Riyadh 11426, Saudi Arabia; 4 Radiological Sciences Program, College of Applied Medical Sciences, King Saud bin Abdulaziz University for Health Sciences, Riyadh 11426, Saudi Arabia

**Keywords:** artificial intelligence, age estimation, deep learning, forensics, machine learning, panoramic radiographs

## Abstract

Automatic age estimation has garnered significant interest among researchers because of its potential practical uses. The current systematic review was undertaken to critically appraise developments and performance of AI models designed for automated estimation using dento-maxillofacial radiographic images. In order to ensure consistency in their approach, the researchers followed the diagnostic test accuracy guidelines outlined in PRISMA-DTA for this systematic review. They conducted an electronic search across various databases such as PubMed, Scopus, Embase, Cochrane, Web of Science, Google Scholar, and the Saudi Digital Library to identify relevant articles published between the years 2000 and 2024. A total of 26 articles that satisfied the inclusion criteria were subjected to a risk of bias assessment using QUADAS-2, which revealed a flawless risk of bias in both arms for the patient-selection domain. Additionally, the certainty of evidence was evaluated using the GRADE approach. AI technology has primarily been utilized for automated age estimation through tooth development stages, tooth and bone parameters, bone age measurements, and pulp–tooth ratio. The AI models employed in the studies achieved a remarkably high precision of 99.05% and accuracy of 99.98% in the age estimation for models using tooth development stages and bone age measurements, respectively. The application of AI as an additional diagnostic tool within the realm of age estimation demonstrates significant promise.

## 1. Introduction

Age plays a crucial role in defining a person’s identity [1]. The pursuit of accurate age estimation methods has persisted throughout time. Whether for living or deceased individuals, the need for reliable age estimation remains significant in various scenarios. There are different approaches to determining someone’s age, which include considering their chronological age, skeletal age, and dental age [2]. Chronological age refers to the length of time that has passed since birth and is the primary way of defining age [3].

Chronological age, alongside biological sex and ethnicity, is a crucial factor in anthropological and forensic studies [4]. Estimating chronological age has been successfully performed by assessing the development of bones. Various skeletal parts, such as the pubic symphysis, auricular surface, and sternal ribs, have been utilized for this purpose. It should be noted that there is not one specific method based on bone development that consistently outperforms others, as the effectiveness of each method depends on numerous factors [5].

Dental maturity is a highly dependable approach for estimating chronological age in criminal, forensic, and anthropological contexts and has the ability to serve as a reliable indicator of age [6,7,8]. Teeth are frequently utilized in age estimation due to their less susceptible nature to external influences, such as genetics or environment [9]. Due to their highly mineralized structure, teeth are resistant to decomposition after death and can withstand flames, alkalis, and acids [10]. While bones may degrade over time, teeth can be preserved for extended periods and are therefore a dependable method of identification in emergency scenarios [11,12].

A blend of techniques—visual, radiographic, chemical, and histological—are utilized for determining dental age. Visual assessment relies on tracking the succession of tooth emergence and functional transitions that accompany aging, like wear and alterations in tooth hue. Radiographic scans offer insight into the developmental stage of teeth, from the inception of mineralization to crown shaping and root tip maturation. Biochemical techniques help to identify changes in ion levels as an individual ages. Histological methods involve preparing tissues for thorough microscopic analysis [8,13,14]. Morphological and radiographic techniques such as Schour and Massler’s method, Demirjian’s method, and Kvaal’s method prove to be effective in determining age in living individuals who are in their teenage and adult years. When it comes to deceased individuals, histological and biochemical techniques like Gustafson’s and Johanson’s method, the Bang and Ramm method, aspartic acid racemization, and the cemental annulation technique come into play for accurately determining age [1,15].

Dental age estimation relies on two distinct methods: assessing the timing of tooth eruption and analyzing the progression of dental maturity stages. The latter, dental maturity, is deemed more dependable due to its high heritability, low coefficient variation, and autonomy from external factors such as nutrition, hormones, and environmental influences [7,16]. Dental radiographic records can help determine a person’s age by assessing different characteristics. These include jaw bone development, tooth germ appearance, stage of tooth crown completion and eruption, extent of deciduous teeth resorption, measurement of open apices in teeth, size of pulp chamber and root canals, formation of secondary dentin, tooth-to-pulp ratio, and development and structure of the third molar [17,18].

Estimating dental age is a complicated task, as teeth come in all shapes and sizes, making it a unique challenge. The complexities and variations within and among individuals further complicate the process [19]. As people become older, they experience changes like reduced alveolar bone levels and altered pulp-to-tooth ratios. However, using only direct measurements of the first molar may result in a significant margin of error of 8.84 years [19]. Moreover, previous methods of age estimation in dentistry have been limited, focusing only on specific aspects of teeth and often resulting in large error margins. Since existing age estimation methods are prone to errors and bias, we hypothesized that an improvement in accuracy could be achieved by removing subjective elements and automating the process. There have been continuous efforts to enhance the precision of AI-powered age estimations, such as utilizing deep learning algorithms, over the past ten years [5].

Dental radiographs have been utilized to demonstrate the dependability of convolutional neural networks (CNNs) for a range of dental ailments like dental caries [20], periodontal disease [21], odontogenic cysts and tumors [22], and conditions affecting the maxillary sinus and temporomandibular joints [23,24]. CNNs have an advantage over traditional individual feature-based techniques as they can conduct end-to-end learning and automatically extract relevant features from raw data without human intervention. They do not need human-engineered techniques, so an AI system could greatly reduce the work of human interpreters or observers in predicting dental age [25]. Additionally, since CNNs generate a comprehensive feature set from data on their own, they perform well with large datasets. Therefore, this systematic review was carried out to assess and report on the performance of AI models developed for automated age estimation from dento-maxillofacial radiographs.

## 2. Materials and Methods

To ensure the quality of the methodology, the authors adhered to the diagnostic test accuracy criteria specified in the Preferred Reporting Items for Systematic Reviews and Meta-Analyses Extension (PRISMA-DTA) [26]. This systematic review protocol is registered in PROSPERO with registration number CRD42024528182. The search for literature was guided by the PICO (Problem/Patient, Intervention/Indicator, Comparison, and Outcome) criteria detailed in Table 1.

### 2.1. Search Strategy

We utilized an array of reputable databases, such as PubMed, Scopus, Embase, Cochrane, Web of Science, Google Scholar, and the Saudi Digital Library, to conduct a digital search for data. Our comprehensive search encompassed the years 2000 to 2024. To search for articles in electronic databases several key terms were used: artificial intelligence, age, age estimation, chronological age, precise age, age prediction, age detection, age evaluation, age assessment, dental age, age classification, tooth staging, tooth parameter, bone parameters, tooth development, convolutional neural network, automated, machine learning, deep learning, X-rays, dental radiographs, panoramic radiographs, and forensics. Further to that, we used Boolean operators (AND, OR) and applied English language filters. To supplement our electronic search, we also manually scrutinized applicable research publications and their citations. This process included inspecting the reference lists of previously gathered articles in the college library. The search was carried out by two separate authors who were specifically trained to carry out the same task.

### 2.2. Study Selection

Article selection was based on how relevant articles were to the field of study, alongside the allure of their titles and abstracts. Two researchers (S.B.K. and F.B.) conducted the search process independently, resulting in a bounty of 580 articles initially considered, with 578 discovered through the electronic search and 2 found through manual exploration. To safeguard against duplicity, two additional team members conducted a thorough inspection, purging 387 replicates. The remaining trove of 193 manuscripts underwent a meticulous assessment to ascertain their eligibility.

### 2.3. Eligibility Criteria

The papers selected for this comprehensive review had to adhere to specific guidelines: (a) original works exploring AI; (b) inclusion of quantitative data for examination and analysis; and (c) clear references to the data enabling evaluation of AI-based models. For the study design to be included in this review, no limitations were imposed. Articles not delving into AI innovation, conference papers never published or that were only available online, unpublished works, articles lacking full-text availability, pilot studies, and those not in English were excluded.

### 2.4. Data Extraction

**Figure 1:**
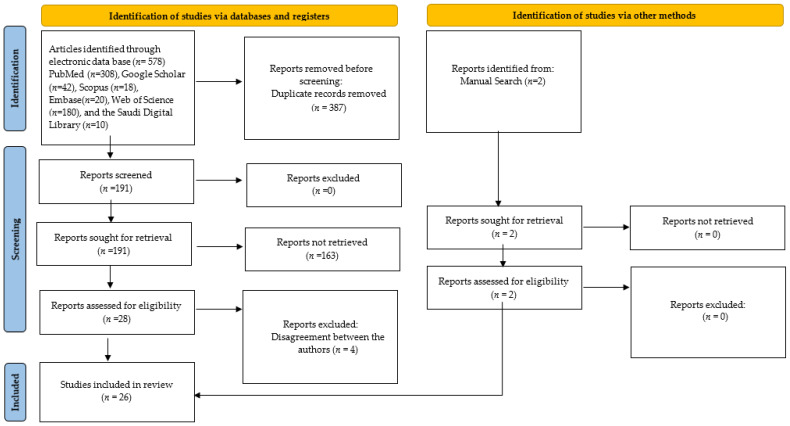
PRISMA 2020 flow diagram for new systematic reviews including searches of databases, registers and other sources.

After evaluating the selected papers based on their titles and abstracts and removing duplicates, the authors thoroughly examined the full texts. Following this evaluation, the count of articles meeting the criteria for inclusion in the systematic review dwindled to 28. To uphold impartiality, the publications’ journal names and author details were expunged, allowing two impartial reviewers (M.A. and A.S.), unconnected to the initial search, to appraise them. Pertinent data from the chosen papers were meticulously extracted and inputted into a Microsoft Excel document, encapsulating particulars on writers, publication dates, research goals, AI algorithm varieties employed, and the data for model training, validation, and testing. Results, findings, and recommendations from the research were also recorded. Disagreements regarding the inclusion of four articles arose due to insufficient evidence supporting their results and conclusions. After consultation with another qualified author (A.F.), these four articles were excluded. As a result, a total of 26 articles were carefully curated and meticulously examined in this systematic review, as shown in Figure 1. These 26 articles were deemed worthy of consideration for inclusion and were subjected to a rigorous evaluation.

The papers were scrutinized for quality using QUADAS-2 [27], which delved into various aspects of research design and reporting, including patient selection, index test, reference standard, flow, and timing. This evaluation sought to gauge the applicability of the data across diverse clinical settings and patient cohorts, while pinpointing possible sources of bias. Two reviewers showed substantial agreement, with an 82% level of agreement measured by Cohen’s kappa.

## 3. Results

After an in-depth analysis of 26 articles, qualitative data were extracted. Most of the articles were published in the last four years, indicating a rising trend in articles focusing on the implementation of AI models for tooth numbering and detection.

### 3.1. Qualitative Data of the Studies

AI has primarily been used for automated age estimation by analyzing tooth development stages [28,29,30,31,32,33,34,35,36,37,38,39,40,41,42,43,44,45,46,47], tooth and bone parameters [48,49,50], bone age measurements [51], and pulp–tooth ratio [52,53]. We gathered data from the studies included, but due to the varied data samples used to assess AI model performance, a meta-analysis could not be conducted. The heterogeneity in the extracted data stemmed from the diverse types of data samples utilized in evaluating the AI models’ performance. Therefore, this systematic review only provides descriptive data from the studies included, as shown in Table 2.

### 3.2. Study Characteristics

The study characteristics decoded comprised details regarding the authors; year of publication; research goals; AI model development algorithms employed; training, validation, and testing data sources; model accuracy assessment; research findings; and any guidance offered by the authors.

### 3.3. Outcome Measures

Efficiency in task execution was evaluated by employing different metrics, such as measurable or predictive outcomes including receiver operating characteristic (ROC), area under the curve (AUC), accuracy, sensitivity, specificity, precision, recall, F-measure, mean absolute error (MAE), root mean squared error (RMSE), R squared (*R*^2^), and root mean squared percentage error (RMSPE).

### 3.4. Risk of Bias Assessment and Applicability Concern

**Figure 2:**
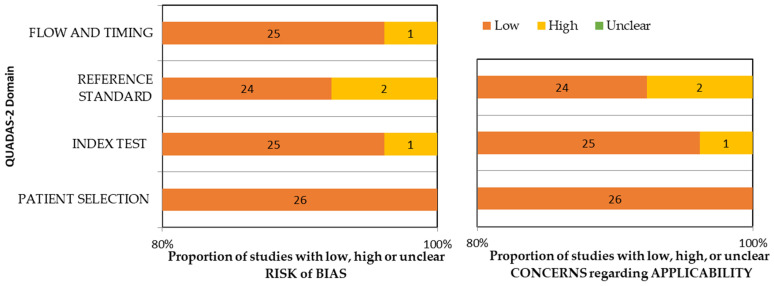
QUADAS-2 assessment of the individual risk of bias domains and applicability concerns.

The evaluation of study quality and risk of bias was conducted using the QUADAS-2 assessment tool (Table S1). All studies employed patient-derived secondary information in the form of dento-maxillofacial radiographs as the input for the CNNs, presuming randomization and non-randomization to be equally dispersed in primary studies. The patient-selection domain was considered to have no risk of bias. The standardized methods for entering data into AI technology helped mitigate bias in the flow and timing domain. Nevertheless, two of the studies (15.38%) [37,46] failed to clearly delineate the reference standard employed, giving rise to inherent bias concerns in the index test, reference standard, flow, and timing domains. Another (7.69%) study [46] relied on notations from solitary observations as a gold standard, culminating in a high risk of bias with respect to index tests. Despite the above-mentioned issues, both research arms exhibited minimal risk of bias in all the studies considered. The risk of bias evaluation and applicability concerns in the studies analyzed are presented in Table S1 and Figure 2.

### 3.5. Assessment of Strength of Evidence

The certainty of evidence was evaluated using the Grading of Recommendations Assessment Development and Evaluation (GRADE) technique [54]. There are four levels of certainty: very low, low, moderate, and high. This is determined by assessing five factors: risk of bias, inconsistency, indirectness, imprecision, and publication bias. According to the assessment, the included papers demonstrated a high level of certainty of evidence, as shown in Table 3.

The certainty of the studies included in this systematic review was evaluated using the Grading of Recommendations Assessment Development and Evaluation (GRADE) approach. Inconsistency, indirectness, imprecision, risk of bias, and publication bias were the five domains that determine the certainty of evidence and can be categorized as very low, low, moderate, or high evidence. The overall certainty of evidence from the included studies in this review was found to be high.

## 4. Discussion

Determining age is crucial in various areas, like in forensic science for identifying individuals in various situations like mass casualties and criminal cases [55]. It also helps to verify the ages of athletes and immigrants to uphold equal rights and fairness [56]. Additionally, it aids in planning orthodontic and pediatric treatments by predicting jaw growth spurts [57]. To accurately estimate age, it is essential to assess sexual characteristics, bone development, and tooth development [12].

Chronological age can be estimated using three main categories: laboratory-guided molecular biology studies, dental indicators, and bone markers [50]. Dental age assessment involves comparing the developmental stages of both temporary and permanent teeth with dental development charts created by various researchers [50]. Researchers have established different scales based on the developmental stages of both permanent and temporary teeth observed in radiographs. The age of 14, when the permanent second molars erupt, marks the conclusion of the childhood and mixed dentition phase and serves as a reliable method in age estimation [37]. Two conventional methods that are commonly used for age determination are the ‘Atlas method’ and ‘Demirjian’s method’. The former compares radiographic dental development (mineralization) with published standards, and the latter is a scoring method that involves scoring the development of seven left lower mandibular teeth in eight categories (A–H) [58].

Even though these manual methods have been correctly utilized in various groups, there are still specific drawbacks in clinical settings, such as the subjectivity of the technique and potential bias in measurement. Additionally, these procedures can be tedious and time-consuming [31]. The conventional approach to dental age estimation using image processing involves a series of manual procedures, including segmentation, feature extraction, image pre-processing, classification, and regression. Each of these steps carries a risk of errors and can introduce variability in the final result. For instance, bone images obtained from radiography scans may differ between dry and wet conditions, even within the same age group [51].

Deep learning methods are known as end-to-end learning-based approaches, where deep neural networks like convolutional neural networks can directly process input images and generate the desired output without the need for intermediate steps like segmentation and feature extraction [29]. Thus, automated dental age estimation is very much essential in order to improve the accuracy and repeatability of age estimation [7]. Hence, this systematic review was undertaken to assess the development and performance of AI models in automated age estimation.

### 4.1. Effectiveness of AI in Automated Age Estimation Using Tooth Development Stages

Dental radiological techniques for age assessment typically involve parameters like tooth development stages, tooth eruption, open apices of teeth, development of jaw bones, and pulp–tooth ratio. Tooth development is more commonly used than eruption in age assessment as the latter can be affected by external factors, whereas formation is a continuous, cumulative, and advancing process [59]. Out of the studies reviewed, a total of 20 have delved into the application of AI for estimating age based on tooth development stages. The study conducted by Mulla et al. [29] achieved the highest accuracy of 98.8% and precision of 99.05% out of all other AI models in age estimation. Their approach was assessed using various performance metrics on a dataset containing 1429 dental X-ray images and indicated that features based on AlexNet outperformed those based on ResNet. Furthermore, the k-NN classifier demonstrated superior performance across different metrics when compared to other classifiers [29].

It was observed in our review that the major drawbacks associated with traditional age estimation methods were underestimation and overestimation of age. It was found that the Chaillet and Demirjian method underestimated the dental age of Malaysian Chinese individuals in the study conducted by Bunyarit SS et al. [28]. Therefore, a population-specific predictive model was created using an artificial neural network–multilayer perceptron (ANN-MLP) to improve the accuracy of age estimation. The discrepancy between chronological age (CA) and dental age (DA) was much lesser (−0.05 ± 0.92 years for boys and −0.06 ± 1.11 years for girls) when utilizing the ANN-MLP networking model. In contrast to this, Galibourg et al. [30] reported that Demirjian’s and Willems’ methods, both overestimated the age, Demirjian’s by a mean of 257 days and Willems’ by 80 days, and affirmed that machine learning methods outperformed traditional approaches for age estimation using radiographic dental staging from childhood to early adulthood. These findings align with a meta-analysis which indicated that Demirjian’s method tends to overestimate females’ ages by 0.65 years and males’ ages by 0.60 years on average [60].

A dental age estimation model that is fully automated, with no human involvement, outperformed one that depended on manually defining features. The automated model achieved a mean absolute error (MAE) of 0.83 years, which was half of that of the manual model. This autonomous method might reveal previously unrecognized age-related features, thereby improving the model’s overall performance as reported by Han M et al. [36].

In another study conducted by Kumagai et al. [42], it was found that the accuracy of the conventional method using the internal test set was slightly better compared to the AI models. The difference in mean absolute error was less than 0.21 years, and the root mean square error was less than 0.24 years. The discrepancies between the conventional methods and AI models were around 44 to 77 days with mean absolute error and 62 to 88 days with root mean square error. While the conventional methods showed a slight edge in accuracy in this research, it is uncertain whether this small difference has significant clinical or practical relevance. These findings suggest that dental age estimation using AI models can be done with nearly the same precision as the conventional method.

Shen et al. [34] conducted a study on estimating the dental age of seven permanent teeth in the left mandible in Eastern Chinese individuals aged 5 to 13 years. They used the Cameriere method for age estimation and compared it with linear regression, support vector machine (SVM), and random forest (RF) models. Their results showed that all three AI models had higher accuracy than the conventional method. The improved accuracy could be due to including younger participants in the study sample. As age estimation becomes more precise with the increasing number of developing teeth, the presence of younger subjects in the study could lead to higher accuracy in the derived age estimation method [61].

### 4.2. Effectiveness of AI in Automated Age Estimation Using Tooth and Bone Parameters

The neural model created in the research conducted by Zaborowicz M et al. [48] demonstrated the lowest prediction errors of 2.34 months in determining the metric age of boys. Specific sets of 21 tooth and bone parameters that were developed as mathematical proportions by the same author were utilized here [62]. The study used panoramic radiographs of people with normal dental development and no systemic illnesses. Cases with root canal treatment or extensive fillings were excluded to improve network construction.

In a different study [49], using the optimal EfficientNet-B5 model, the group of females aged 22 to 31 had the smallest prediction error (MAE 0.96, RMSE 1.52), whereas the group of males aged 52 to 61 had the highest error (MAE 5.12, RMSE 7.03). The discrepancy between estimated and real age increased with age. The dentition, maxillary sinus, mandibular body, and mandibular angle all contributed to age estimation. The class activation mapping results indicated that different anatomical structures were relevant in age groups. Characteristics were predominantly in the teeth in younger age groups (12 to 21 and 22 to 31 years), which is in line with conventional techniques. The emphasis turned to the maxillary sinus upon movement into the middle age groups (ages 32 to 41 and 42 to 51). Mandibular body and mandibular angle were emphasized for older age groups (52–61 and 62–71 years) [49].

In comparison to the ResNet101 network, VGG16 demonstrated superior performance in estimating DA using OPGs on a large scale using teeth and bone parameters according to the study conducted by Wang J et al. [51]. The VGG16 model yielded satisfactory predictions for younger age groups, with an accuracy of up to 93.63% in the 6- to 8-year-old category [50].

### 4.3. Effectiveness of AI in Automated Age Estimation Using Bone Parameters

A novel automated machine learning model for bone age estimation was proposed by Sharifonnasabi F et al. [51]. The current bone age estimation models are mainly in the research phase and have not been widely adopted in the industry. The proposed model achieved high accuracy (99.98%) levels for different age ranges and outperformed existing models. Testing on diverse datasets and races confirmed the superior performance of the HCNN-KNN model, making it a promising tool for bone age measurement [51].

### 4.4. Effectiveness of AI in Pulp–Tooth Ratio

Dental age estimation in adults is based on quantifying age-related morphological changes of teeth, such as the deposition of secondary dentin. Even after the completion of root formation, the odontoblasts remain functional, continuing the production of secondary dentin throughout life. As a result of this physiological process, the dimensions of the pulp chamber gradually change [63]. Various age estimation methods have been developed based on this decrease. This assessment can be definitively performed through non-radiological methods like histological and biochemical approaches. Due to the need for tooth extraction in these methods, they are not suitable for living individuals or situations where tissue collection from human remains is not feasible. Therefore, radiological methods are more easily applicable for dental age estimation. Radiological methods have progressed significantly, enabling the three-dimensional imaging of hard tissues in the jaws [64].

Pulp chamber volumes are utilized in estimating dental age, and these ratios can be analyzed through deep learning. Despite the low performance of the models as reported in a study conducted by Dogan B et al. [53], they represent a different approach. The algorithms performed most accurately for the 18–25 age group compared to other age groups. Exploring different parameters derived from various measuring techniques in CBCT data could aid in developing machine learning algorithms for age classification in forensic scenarios. The measurements should be always taken from the cementum–enamel junction level on the axial section to obtain accurate three-dimensional secondary dentine deposition [53].

A study evaluated the effectiveness of Kvaal’s age estimation method using various ML attribute extraction approaches and algorithms on a population from northeastern Brazil, based on pulp–tooth ratios. The findings suggested a positive outcome for the semantic–radiomic association attribute. Kvaal’s method and ML yielded better results for the male dataset, with ML outperforming the Kvaal method by around 1 year across all analyzed scenarios [52].

## 5. Challenges and Future Considerations in AI

AI models developed for age estimation using dento-maxillofacial radiographs can be applied for various tasks like determining identities of dead people in explosions and bomb blasts, evaluating athletes in competitive sports, judging juvenile delinquencies, clinical and forensic purposes, adopting undocumented children of uncertain ages, handling international refugees, and planning treatment for patients. Despite promising results from studies evaluating the performance of AI models for automated age estimation from dento-maxillofacial radiographs, various factors need to be considered before a definitive conclusion can be reached. The limitations and challenges reported in most of the studies are mainly related to the limited number of datasets and the lack of a good number of previously reported studies for a comparison of the results. Hence, it is necessary to conduct more studies with abundant sample sizes and in diverse populations in order to improve the applicability of this approach. The requirement for abundant data can also be addressed through the application of data augmentation methods. Furthermore, the training datasets need to be precise, reliable, and free from significant errors to ensure optimal performance [65]. Some studies reviewed had used a limited number of dental radiograph datasets compared to the wealth of data utilized in medical AI research. This may lead to the development of AI models that are excessively specialized, potentially skewing results towards overly optimistic outcomes. The essence of this issue lies in the fact that AI algorithms typically require a substantial amount of data for effective generalization to diverse scenarios. Consequently, it is crucial to validate the sample size and possibly conduct statistical analyses to ensure that the findings have broader applicability. The overall findings of the studies included in the paper suggest that AI-based models which include ML and DL display high accuracy and minimal average error and outperform the classical methods applied for age estimation [34,39]. However, the mean error of deep learning techniques is claimed to be somewhat greater than that of machine learning regression approaches, despite the fact that they can save more time in object detection [34]. When applying ML models for age estimation, we should consider individual variability and use additional predictors in order to reduce the variability [66]. Deep learning techniques can perform a more detailed analysis process, where they can work directly on input images and provide the desired output without requiring the completion of intermediate processes like feature extraction and segmentation. However, designing and training a deep neural network is an expensive, time-consuming, and difficult process. Therefore, new approaches are developed that can utilize pre-trained deep neural networks and perform the necessary tasks, and these approaches are termed transfer learning methods [31]. These transformers have made a breakthrough in computer vision. Age estimation models that were developed using transfer learning were more feasible in terms of cost, time spent in developing the model, and performing the task more precisely [31,49].

## 6. Conclusions

This systematic review found that AI models demonstrated superior performance in automatic age estimation utilizing dento-maxillofacial radiographic images with increased accuracy and precision and decreased mean absolute errors. Given this specific situation, AI has the potential to act as a valuable partner in supporting the efforts of dental and forensic professionals by allowing them to handle numerous images simultaneously. However, it is crucial to recognize that the results of AI radiographic analyses are not inherently flawless, as their precision depends on the quality of the training data and the effectiveness of their model’s selection and training procedures. Thus, it remains essential for experts to provide their ultimate interpretation as the final assessment.

## Figures and Tables

**Table 1:** Description of the PICO (P = Population, I = Intervention, C = Comparison, O = Outcome) elements.

Research question	What are the developments and performance of the artificial intelligence models that have been used in age estimation using dento-maxillofacial radiographs?
Population	Patients who underwent dento-maxillofacial radiographs
Intervention	AI-based models developed for age estimation
Comparison	Traditional methods of age estimation, expert opinion, other AI models
Outcome	Accuracy, sensitivity, specificity, precision, recall, receiver, area under the curve (AUC), F measure, mean absolute error (MAE), root mean squared error (RMSE), R squared (*R*^2^), root mean squared percentage error (RMSPE).

**Table 2:** Details of the studies that have used automation-based models for age estimation.

SI No.	Authors	Year of Publication	Study Design	Algorithm Architecture	Objective of the Study	No. of Patients/Images/Photographs for Testing [Datasets]	Study Factor	Modality	Comparison If Any	Evaluation Accuracy/Average Accuracy/Statistical Significance	Results (+) Effective, (−) Non-Effective (N) Neutral	Outcomes	Authors Suggestions/Conclusions
1.	Bunyarit SS et al. [28]	2018	Retrospective cross-sectional study	ANN	To investigate the applicability of Chaillet and Demirjian’s scores for the age estimation of Malaysian Chinese children and adolescents	1228 DPRs	Tooth development stages (permanent teeth from central incisor to third molar-left mandible).	DPRs	Chaillet and Demirjian’s method	Accurate estimation of age and the difference between CA and DA was −0.05 ± 0.92 years for boys and −0.06 ± 1.11 years for girls using ANN–MLP networking model.	Effective (+)	Chaillet and Demirjian’s method underestimated the DA. Population-specific prediction model was developed using the ANN–MLP networking model.	These ethnic-specific data can be used to estimate the DA of Malaysian Chinese children and adolescents in both clinical and forensic applications.
2.	Mualla N et al. [29]	2020	Observational study	CNN	To implement automated dental age estimation using transfer learning	1429 X-ray images	Tooth development stages	X-rays	ResNet	AlexNet (KNN classifier) Accuracy 98.8% Specificity 99.18 Precision 99.005 Recall 99.98 F-measure 98.99 ResNet Accuracy 98.8% Specificity 99.818 Precision 99.149 Recall 99.062 F-measure 99.104	Effective (+)	AlexNet-based features were better than the ResNet-based features.	Transfer learning has proved its effectiveness in many machine learning and object recognition problems. This method needs to be evaluated more using a larger dataset and other classification models.
3.	Galibourg A et al. [30]	2021	Comparative study	ML	To compare the predictive performance of ten machine learning algorithms for dental age estimation in children and young adults using left permanent mandibular teeth and third molars	3570 OPGs	Tooth development stages (seven permanent mandibular teeth and four third molars)	OPG	Age estimation methods by Demirjian et al. and Willems et al.	AI method had a mean absolute error (MAE) under 0.811 years. With Demirjian’s and Willems’ methods, the MAE was 1.107 and 0.927 years,	Effective (+)	WILL method was significantly more accurate than DEM, and all ML methods were more accurate than the best reference method.	This study supported the use of ML algorithms instead of using standard population tables.
4.	İsa AT al. [31]	2021	Comparative study	DL models	To estimate the forensic ages of individuals using an automated approach	1332 DPRs Training 962 (85%), testing 170 (15%), and validating 200 (15%)	Tooth development stages (teeth, gingival tissue, and upper jaw)	DPR Images	InceptionV3 DenseNet201 EffcientNetB4MobileNetV2 VGG16, and ResNet50V2	MAE = 3.13, RMSE =4.77, and correlation coefficient *R*^2^ was 87%.	Effective (+)	Modified InceptionV3 model delivered results faster and estimated ages more precisely compared to others.	This method proved to be potentially dependable and practical ancillary equipment in forensic sciences and dental medicine.
5.	Wallraff S et al. [32]	2021	Comparative study	CNN	To automate age estimation of adolescents using a supervised regression-based deep learning method	14000 DPRs 10,000 for training, 2500 for validation, and 244 for testing	Tooth development stages	DPR	Senior physician for oral and maxillofacial surgery and three dental students specially trained in age estimation	MAE of 1.08 years and error rate (ER) of 17.52%.	Effective (+)	This method achieved better results than manual estimation methods used by clinical experts.	This method will be useful in age estimation compared to manual methods.
6.	Kim S et al. [33]	2021	Comparative study	CNN	To estimate age group by incorporating a CNN using dental X-ray image patches of the first molars extracted via panoramic radiography	DPRs of 1586 individuals Training: 1078 Validation: 190 Test: 318	Tooth development stages (permanent first molars of both arches)	Panoramic radiographs	Subgroup comparison	The accuracy of the tooth-wise estimation was 89.05 to 90.27%. AUC scores ranged from 0.94 to 0.98 for all age groups.	Effective (+)	Study demonstrated the suitability of CNNs for accurately estimating the age groups of both the maxillary and mandibular first molars.	The prediction accuracy and heat map analyses support that this AI-based age-group determination model is plausible and useful.
7.	Shen S et al. [34]	2021	Retrospective study	ML	To utilize seven lower left permanent teeth alongside three models (RF, SVM, and LR) based on the Cameriere method for predicting children’s dental age and assess their performance against Cameriere age estimation	748 OPGs Training: 598 (80%) Test: 150 (20%)	Tooth development stages (7 lower left permanent teeth)	OPG	Traditional Cameriere formula	ME, MAE, MSE, and RMSE values of the SVM model (0.004, 0.489, 0.392, and 0.625, respectively) and the RF model (−0.004, 0.495, 0.389, and 0.623, respectively) had the highest accuracy.	Effective (+)	The research showed that the ML models have better accuracy than the traditional Cameriere formula.	All tested machine learning methods were significantly more accurate than the two Cameriere formulas for all metrics.
8.	Milošević D et al. [35]	2022	Comparative study	CNN	To explore the applicability of deep learning in chronological age estimation using panoramic dental X-rays	4035 DPRs training/validation/test size is 80%/10%/10%	Tooth development stages	DPR	State of art methods	Mean absolute error of 3.96 years, a median absolute error of 2.95 years, and an *R*^2^ of 0.8439.	(+) Effective	This research showcases the effectiveness of automated deep learning in dental imaging for precise age estimation.	The proposed approach attains the lowest estimation error in the literature for adult and senior subjects.
9.	Hann et al. [36]	2022	Cross-sectional, descriptive, analytical study.	CNN	To assess the accuracy of a machine learning model for precise age estimation with or without relying on human interference	10,257 OPGs Training set (80%), validation set (10%), and test set (10%).	Tooth development stages (8 permanent teeth of left mandible)	OPG	ADSE model based on specified manually defined features	MAE of the ADAE model is 0.83 years, being reduced by half that of the MDAE model. ADSE model for stage classification is 0.17 stages; its accuracy in dental age estimation is unsatisfactory.	(+) Effective	The ADSE model slightly improves accuracy with manually defined features and enhances evaluation efficiency. In contrast, the ADAE model, utilizing CNN, significantly boosts accuracy and efficiency without human intervention.	Fully automated feature extraction in a deep learning model without human interference performs better in dental age estimation, prominently increasing the accuracy and objectivity.
10.	Baydoğan MP et al. [37]	2022	Observational study	CNN	To estimate age by deep learning using dental panoramic radiographs	627 OPGs Training (70%) Test (30%)	Tooth development stages	OPG	Dentist	84% accuracy, 85% F-score, and 76% sensitivity values were reached using the Alexnet architecture and k-nearest neighbor (k-NN) algorithm.	(+) Effective	The proposed system will ensure age determination in less time and abate the cost compared to the traditional age determination method.	The study will support dentists in the clinical environment and can be used in education
11.	Pintana P et al. [38]	2022	Observational study	CNN	To develop and implement a fully automated system for estimating dental age utilizing the ACF detector and deep learning methodologies	1000 OPGs Training: 800 Testing: 200	Tooth development stages (lower left third molars)	OPG	Demirjian’s method	Localized the lower left mandibular third molar automatically with 99.5% accuracy and achieved 83.25% classifcation accuracy using the transfer learning strategy with the Resnet50 network.	(+) Effective	ACF detector and CNN model successfully localized third molars and identified the developmental stages automatically in order to estimate the age of the subjects.	The proposed method can be applied in clinical practice as a tool that helps clinicians reduce the time and subjectivity for dental age estimation.
12.	Saric et al. [39]	2022	Observational study	ANN	To decide on the most desirable machine learning for dental age estimation based on buccal bone level	150 CBCTs	Tooth development stages (lower seven mandibular teeth)	CBCT	Conventional ML models	Random forest classifier provided the greatest result with a correlation coefficient of 0.803 and a mean absolute error of 6.022.	(+) Effective	*RF* proved to be the best algorithm in our study, providing the most acceptable result for age estimation, using the three most important attributes.	We have also shown that considering sinus-related features can be a significant addition to the databases.
13.	Shen et al. [40]	2022	Comparative study	ML	To compare of the accuracy of the Cameriere and Demirjian methods of dental age estimation using ML simultaneously	748 OPGs Training: 80% Testing: 20%	Tooth development stages (seven mandibular teeth)	OPG	Demirjian method or Cameriere European formula	KNN model based on the Cameriere method had the highest accuracy (ME = 0.015, MAE = 0.473, MSE = 0.340, RMSE = 0.583, *R*^2^ = 0.94),	(+) Effective	KNN model based on the Cameriere method was able to infer dental age more accurately in a clinical setting.	ML can be used for dental age estimation in a larger geographical area and over a larger age range.
14.	Wang et al. [41]	2022	Comparative study	CNN	To estimate chronological ages by using DENSEN for different age groups	1903 OPGs	Tooth development stages	OPG	Bayesian CNN Net and DANet	DENSEN produced MAEs of 0.6885, 0.7615, 1.3502, and 2.8770 for children teens, young adults, and adults, respectively.	(+) Effective	DENSEN has lower errors for the adult group. The proposed model is memory-compact, consuming about 1.0 MB of memory overhead.	This approach required less laboratory work compared with existing methods.
15.	Kumagai A et al. [42]	2023	Comparative study	ML	To validate data-mining-based dental age estimation by comparing its accuracy and classification performance at 18-year thresholds against conventional methods	2657 DPRs Training: 900 Test sets: 857	Tooth development stages (second and third molars of both jaws)	DPRs	Conventional method	The accuracy of the conventional method with the internal test set was slightly higher than that of the data mining models, with a slight difference (mean absolute error< 0.21 years, root mean square error< 0.24 years).	Neutral (N)	The threshold was also similar between the conventional method and the data mining models.	This method proved that conventional methods can be replaced by data mining models in forensic age estimation using second and third molar maturity of Korean juveniles and young adults.
16.	Yeom HG et al. [43]	2023	Comparative study	CNN	To estimate chronological age using a hybrid method based on ResNet 50 and ViT	9663 DPRs Training: 5861 Validation: 1916 Test data: 1886	Tooth development stages	DPRs	ResNet50 or ViT.	Significant improvements were observed in both MAE and RMSE values across all network models (ResNet50, ViT, and Hybrid	(+) Effective	The age estimation model designed using the hybrid method performed better than those using only ResNet50 or ViT.	This model can perform better and be used effectively in the clinical field.
17.	Kahm SH et al. [44]	2023	Comparative study	DL	To evaluate the efficiency of an AI model by applying the entire panoramic image for age estimation	27,877 DPRs Training: 13,220 Validation: 1653 Test data: 1653	Tooth development stages	DPRs	Two experienced dentists	Incorporating ± 3years of deviation, the accuracy of type 1 and 2 was 0.2716, 0.7323, respectively; and the F1 score was 0.1709 and 0.6437, respectively.	(+) Effective	The study showed significant accurate diagnosis in type 2 grouping with ±3years of deviation in both DN and WRN models.	In the future, the application of AI is expected to assist humans in clinical and dento-maxillofacial radiology fields.
18.	Aljameel S et al. [47]	2023	Comparative study	CNN	To predict dental age using AI model	529 DPRs 423 (80% for training) 106 (20% for testing)	Tooth development stages (7 left permanent teeth and 3 molars)	DPRs	Three dentists	Xception model had the best performance, with an error rate of 1.417 for the 6–11 age group.	(+) Effective	Xception model had the best performance, with an error rate of 1.417 for the 6–11 age group.	The proposed model can assist dentists in determining the appropriate treatment for patients based on their DA rather than their chronological age.
19.	Rin Kim et al. [46]	2023	Observational study	CNN	To classify the age group using deep neural network when precise age information is not given	10023 DPRs	Tooth development stages	DPRs	NM	The accuracies were 53.846% with a tolerance of ±5 years, 95.121% with ±15 years, and 99.581% with ±25 years, which means the probability of the estimation error being larger than one age group is 0.419%.	(+) Effective	This study confirmed the potential possibility of age estimation using AI in terms of clinical aspects of oral care, as well as forensic medicine, by determining the difference between the actual age and predicted age using panoramic radiographic images, which can be used to evaluate the overall oral conditions.	This study has the potential to be used as oral health education material using the difference between the actual age and the predicted age through AI in dental clinics.
20.	Murray J et al. [47]	2024	Observational study	CNN	To apply AI in determination of legal age	4003 DPRs Training: 80% Testing: 20%	Tooth development stages (third molars)	DPRs	Experts	Of the subjects over 18 years of age, 88% were correctly identified, and 87.0% of subjects under the age of majority were similarly predicted.	(+) Effective	AI-based methods could improve courtroom efficiency, stand as automated assessment methods, and contribute to our understanding of biological aging.	The present model may be used as an automated assessment tool for identifying legal age. The weightings generated by this architecture can also help researchers identify which patterns are most significant for understanding this challenging age group.
21.	Zaborowicz M et al. [48]	2022	Observational study	DL	To utilize deep learning neural models for accurate assessment of chronological age in children and adolescents based on tooth and bone parameters	619 OPGs	Tooth and bone parameters	OPG	Radial basis function networks	The MAE error of the produced models, depending on the learning set used, is between 2.34 and 4.61 months, while the RMSE error is between 5.58 and 7.49 months. The correlation coefficient *R*^2^ ranges from 0.92 to 0.96.	(+) Effective	The conducted research indicates that neural modeling methods are an appropriate tool for determining the metric age based on the developed proprietary tooth and bone indices.	The initial iteration of learning the network with all developed metrics already yields high-quality deep neural models. It is advisable to construct deep neural networks using the indicators from the initial research stage.
22.	MU CC et al. [49]	2022	Comparative study	DL	To asess the accuracy of transfer learning models for age estimation from panoramic radiographs of permanent dentitions of patients	3000 DPRs Training: 2400 Validating: 300 Test set: 300	Tooth and bone parameters (teeth, maxillary sinus, and mandibular angle)	DPRs	ResNet VggNet DenseNet	MAE and RMSE of EfficentNet–B5 were 2.83 and 4.49, respectively	(+) Effective	This method of transfer learning proves to be applicable for age estimation utilizing panoramic radiographs.	This model can be used for age estimation with panoramic radiographs.
23.	Wang J et al. [50]	2023	Comparative study	CNN	To investigate the possibility of using AI-based methods for age estimation in an eastern Chinese population	9586 OPGs Training: 70% Testing: 30%	Tooth and bone parameters (molars, maxillary sinus, and nasal septum)	OPGs	ResNet101	Accuracy of VGG16 model = 93.63%. Accuracy of ResNet101 network = 88.73%.	(+) Effective	VGG16 outperformed ResNet101 in terms of DA prediction performance.	CNNs such as VGG16 hold great promise for future use in clinical practice and forensic sciences.
24.	Sharifonnasabi F et al. [51]	2022	Comparative study	CNN	To evaluate the accuracy of a hybrid HCNN-KNN model in age estimation	1922 DPRs Training: 80% Testing: 20%	Bone age measurement	OPG	ResNet, CNN, GoogLeNet Inception	Successfully estimated the age in classified studies of - year-old, 6 months, 3 months, and 1-month-old cases with accuracies of 99.98, 99.96, 99.87, and 98.78 respectively.	(+) Effective	The evaluation of our model on a diverse dataset confirms its superior performance.	The benchmarking with current existing models also showed that the HCNN-KNN model is the best model for bone age measurement.
25.	Pereira de Sousa et al. [52]	2023	Comparative study	ML	To assess and compare age estimation on panoramic radiography using the Kvaal method and machine learning	554 DPRs Training: 85% Testing: 15%	Pulp–tooth ratio	DPRs	Kaval method	ML (MAE: 4.77 presented higher age estimation precision than the Kvaal method (MAE: 5.68).	(+) Effective	ML classifiers can improve age estimation when assessing panoramic radiography using the Kvaal method.	The use of ML on panoramic radiographs can improve age estimation.
26.	Dogan B et al. [53]	2024	Observational study	ML	To use ML algorithms to evaluate the efficacy of pulp/tooth area ratio (PTR) in cone-beam CT (CBCT) images to predict dental age classification in adults	236 CBCT Training: 70% Testing: 30%	Pulp/tooth area ratio	CBCT	CART SVM	The models’ highest accuracy and confidence intervals were found to belong to the RF algorithm.	Neutral (N)	The models’ performances were found to be low.	The models were found to be low in performance but were considered as a different approach.

Notes: ML = machine learning; DL = deep learning; ANN = artificial neural networks; CNN = convolutional neural networks; DPRs—digital panoramic radiographs; OPGs—orthopantomographs; CT—computed tomography; CBCT—cone-beam computed tomography; MAE—mean absolute error; RMSE—Root Mean Squared Error; *R*^2^—R squared.

**Table 3:** Assessment of strength of evidence.

Outcome	Inconsistency	Indirectness	Imprecision	Risk of Bias	Publication Bias	Strength of Evidence
Application of AI in automated age estimation using tooth development stages [28,29,30,31,32,33,34,35,36,37,38,39,40,41,42,43,44,45,46,47]	Not Present	Not Present	Not Present	Present	Not Present	⨁⨁⨁◯
Application of AI in automated age estimation using tooth and bone parameters [48,49,50]	Not Present	Not Present	Not Present	Not Present	Not Present	⨁⨁⨁⨁
Application of AI in automated age estimation using bone age measurements [51]	Not Present	Not Present	Not Present	Not Present	Not Present	⨁⨁⨁⨁
Application of AI in automated age estimation using pulp–tooth ratio [52,53]	Not Present	Not Present	Not Present	Not Present	Not Present	⨁⨁⨁⨁

⨁⨁⨁⨁—high evidence; ⨁⨁⨁◯—moderate evidence.

## Data Availability

Not applicable.

## Supplementary Materials

The following supporting information can be downloaded at: https://www.mdpi.com/article/10.3390/diagnostics14111079/s1, Table S1: Risk of bias and applicability concerns.

## References

[1] Limdiwala P., Shah J. (2013). Age Estimation by Using Dental Radiographs. J. Forensic Dent. Sci..

[2] Willems G., Moulin-Romsee C., Solheim T. (2002). Non-Destructive Dental-Age Calculation Methods in Adults: Intra- and Inter-Observer Effects. Forensic Sci. Int..

[3] Maltoni R., Ravaioli S., Bronte G., Mazza M., Cerchione C., Massa I., Balzi W., Cortesi M., Zanoni M., Bravaccini S. (2022). Chronological Age or Biological Age: What Drives the Choice of Adjuvant Treatment in Elderly Breast Cancer Patients?. Transl. Oncol..

[4] Franklin D. (2010). Forensic Age Estimation in Human Skeletal Remains: Current Concepts and Future Directions. Leg. Med..

[5] Vila-Blanco N., Varas-Quintana P., Tomás I., Carreira M.J. (2023). A Systematic Overview of Dental Methods for Age Assessment in Living Individuals: From Traditional to Artificial Intelligence-Based Approaches. Int. J. Leg. Med..

[6] Lewis J.M., Senn D.R. (2010). Dental Age Estimation Utilizing Third Molar Development: A Review of Principles, Methods, and Population Studies Used in the United States. Forensic Sci. Int..

[7] Celik S., Zeren C., Çelikel A., Yengil E., Altan A. (2014). Applicability of the Demirjian Method for Dental Assessment of Southern Turkish Children. J. Forensic Leg. Med..

[8] Uzuner F.D., Kaygısız E., Darendeliler N. (2017). Defining Dental Age for Chronological Age Determination. Post Mortem Exam. Autops..

[9] Willems G. (2001). A Review of the Most Commonly Used Dental Age Estimation Techniques. J. Forensic Odonto-Stomatol..

[10] Reesu G.V., Augustine J., Urs A.B. (2015). Forensic Considerations When Dealing with Incinerated Human Dental Remains. J. Forensic Leg. Med..

[11] Stavrianos C., Mastagas D., Stavrianou I., Karaiskou O. (2008). Dental Age Estimation of Adults: A Review of Methods and Principles. Res. J. Med. Sci..

[12] Panchbhai A. (2011). Dental Radiographic Indicators, a Key to Age Estimation. Dentomaxillofacial Radiol..

[13] AlQahtani S.J., Hector M.P., Liversidge H.M. (2010). Brief Communication: The London Atlas of Human Tooth Development and Eruption. Am. J. Phys. Anthropol..

[14] Blenkin M., Taylor J. (2012). Age Estimation Charts for a Modern Australian Population. Forensic Sci. Int..

[15] Reppien K., Sejrsen B., Lynnerup N. (2006). Evaluation of Post-Mortem Estimated Dental Age versus Real Age: A Retrospective 21-Year Survey. Forensic Sci. Int..

[16] McKenna C., James H., Taylor J., Townsend G. (2002). Tooth Development Standards for South Australia. Aust. Dent. J..

[17] Liversidge H.M., Smith B.H., Maber M. (2010). Bias and Accuracy of Age Estimation Using Developing Teeth in 946 Children. Am. J. Phys. Anthropol..

[18] Mani S.A., Naing L., John J., Samsudin A.R. (2008). Comparison of Two Methods of Dental Age Estimation in 7–15-Year-Old Malays. Int. J. Paediatr. Dent..

[19] Shah P., Venkatesh R. (2016). Pulp/Tooth Ratio of Mandibular First and Second Molars on Panoramic Radiographs: An Aid for Forensic Age Estimation. J. Forensic Dent. Sci..

[20] Lee J.-H., Kim D.-H., Jeong S.-N., Choi S.-H. (2018). Detection and Diagnosis of Dental Caries Using a Deep Learning-Based Convolutional Neural Network Algorithm. J. Dent..

[21] Chen I.-H., Lin C.-H., Lee M.-K., Chen T.-E., Lan T.-H., Chang C.-M., Tseng T.-Y., Wang T., Du J.-K. (2024). Convolutional-Neural-Network-Based Radiographs Evaluation Assisting in Early Diagnosis of the Periodontal Bone Loss via Periapical Radiograph. J. Dent. Sci..

[22] Yang H., Jo E., Kim H.J., Cha I., Jung Y.-S., Nam W., Kim J.-Y., Kim J.-K., Kim Y.H., Oh T.G. (2020). Deep Learning for Automated Detection of Cyst and Tumors of the Jaw in Panoramic Radiographs. J. Clin. Med..

[23] Serindere G., Bilgili E., Yesil C., Ozveren N. (2022). Evaluation of Maxillary Sinusitis from Panoramic Radiographs and Cone-Beam Computed Tomographic Images Using a Convolutional Neural Network. Imaging Sci. Dent..

[24] Choi E., Kim D., Lee J.-Y., Park H.-K. (2021). Artificial Intelligence in Detecting Temporomandibular Joint Osteoarthritis on Orthopantomogram. Sci. Rep..

[25] Merdietio Boedi R., Banar N., De Tobel J., Bertels J., Vandermeulen D., Thevissen P.W. (2019). Effect of Lower Third Molar Segmentations on Automated Tooth Development Staging Using a Convolutional Neural Network. J. Forensic Sci..

[26] Page M.J., McKenzie J.E., Bossuyt P.M., Boutron I., Hoffmann T.C., Mulrow C.D., Shamseer L., Tetzlaff J.M., Akl E.A., Brennan S.E. (2021). The PRISMA 2020 Statement: An Updated Guideline for Reporting Systematic Reviews. Br. Med. J..

[27] Whiting P.F. (2011). QUADAS-2: A Revised Tool for the Quality Assessment of Diagnostic Accuracy Studies. Ann. Intern. Med..

[28] Bunyarit S.S., Nambiar P., Naidu M., Asif M.K., Poh R.Y.Y. (2022). Dental Age Estimation of Malaysian Indian Children and Adolescents: Applicability of Chaillet and Demirjian’s Modified Method Using Artificial Neural Network. Ann. Hum. Biol..

[29] Mualla N., Houssein E.H., Hassan M.R. (2020). Dental Age Estimation Based on X-ray Images. Comput. Mater. Contin..

[30] Galibourg A., Cussat-Blanc S., Dumoncel J., Telmon N., Monsarrat P., Maret D. (2021). Comparison of Different Machine Learning Approaches to Predict Dental Age Using Demirjian’s Staging Approach. Int. J. Leg. Med..

[31] Atas İ., Özdemir C., Atas M., Dogan Y. (2023). Forensic Dental Age Estimation Using Modified Deep Learning Neural Network. Balk. J. Electr. Comput. Eng..

[32] Wallraff S., Vesal S., Syben C., Lutz R., Maier A. (2021). Age Estimation on Panoramic Dental X-Ray Images Using Deep Learning. Bildverarbeitung für die Medizin 2021.

[33] Kim S., Lee Y.-H., Noh Y.-K., Park F.C., Auh Q.-S. (2021). Age-Group Determination of Living Individuals Using First Molar Images Based on Artificial Intelligence. Sci. Rep..

[34] Shen S., Liu Z., Wang J., Fan L., Ji F., Tao J. (2021). Machine Learning Assisted Cameriere Method for Dental Age Estimation. BMC Oral Health.

[35] Milošević D., Vodanović M., Galić I., Subašić M. (2022). Automated Estimation of Chronological Age from Panoramic Dental X-Ray Images Using Deep Learning. Expert Syst. Appl..

[36] Han M., Du S., Ge Y., Zhang D., Chi Y., Long H., Yang J., Yang Y., Xin J., Chen T. (2022). With or without Human Interference for Precise Age Estimation Based on Machine Learning?. Int. J. Leg. Med..

[37] Baydoğan M.P., Baybars S.C., Tuncer S.A. (2022). Age Detection by Deep Learning from Dental Panoramic Radiographs. Artif. Intell. Theory Appl..

[38] Pintana P., Upalananda W., Saekho S., Yarach U., Wantanajittikul K. (2022). Fully Automated Method for Dental Age Estimation Using the ACF Detector and Deep Learning. Egypt. J. Forensic Sci..

[39] Saric R., Kevric J., Hadziabdic N., Osmanovic A., Kadic M., Saracevic M., Jokic D., Rajs V. (2022). Dental Age Assessment Based on CBCT Images Using Machine Learning Algorithms. Forensic Sci. Int..

[40] Shen S., Yuan X., Wang J., Fan L., Zhao J., Tao J. (2022). Evaluation of a Machine Learning Algorithms for Predicting the Dental Age of Adolescent Based on Different Preprocessing Methods. Front. Public Health.

[41] Wang X., Liu Y., Miao X., Chen Y., Cao X., Zhang Y., Li S., Zhou Q. (2022). DENSEN: A Convolutional Neural Network for Estimating Chronological Ages from Panoramic Radiographs. BMC Bioinform..

[42] Kumagai A., Jeong S., Kim D., Kong H.-J., Oh S., Lee S.-S. (2023). Validation of Data Mining Models by Comparing with Conventional Methods for Dental Age Estimation in Korean Juveniles and Young Adults. Sci. Rep..

[43] Yeom H.-G., Lee B.-D., Lee W., Lee T., Yun J.P. (2023). Estimating Chronological Age through Learning Local and Global Features of Panoramic Radiographs in the Korean Population. Sci. Rep..

[44] Kahm S.H., Kim J.-Y., Yoo S., Bae S.-M., Kang J.-E., Lee S.H. (2023). Application of Entire Dental Panorama Image Data in Artificial Intelligence Model for Age Estimation. BMC Oral Health.

[45] Aljameel S.S., Althumairy L., Albassam B., Alsheikh G., Albluwi L., Althukair R., Alhareky M., Alamri A., Alabdan A., Shahin S.Y. (2023). Predictive Artificial Intelligence Model for Detecting Dental Age Using Panoramic Radiograph Images. Big Data Cogn. Comput..

[46] Kim Y.-R., Choi J.-H., Ko J., Jung Y.-J., Kim B., Nam S.-H., Chang W.-D. (2023). Age Group Classification of Dental Radiography without Precise Age Information Using Convolutional Neural Networks. Healthcare.

[47] Murray J., Heng D., Lygate A., Porto L., Abade A., Manica S., Franco A. (2023). Applying Artificial Intelligence to Determination of Legal Age of Majority from Radiographic. Morphol. Bull. L’association Anat..

[48] Zaborowicz M., Zaborowicz K., Biedziak B., Garbowski T. (2022). Deep Learning Neural Modelling as a Precise Method in the Assessment of the Chronological Age of Children and Adolescents Using Tooth and Bone Parameters. Sensors.

[49] Mu C.C., Li G. (2022). Age Estimation Using Panoramic Radiographs by Transfer Learning. Chin. J. Dent. Res..

[50] Wang J., Dou J., Han J., Li G., Tao J. (2023). A Population-Based Study to Assess Two Convolutional Neural Networks for Dental Age Estimation. BMC Oral Health.

[51] Sharifonnasabi F., Jhanjhi N.Z., John J., Obeidy P., Band S.S., Alinejad-Rokny H., Baz M. (2022). Hybrid HCNN-KNN Model Enhances Age Estimation Accuracy in Orthopantomography. Front. Public Health.

[52] Pereira de Sousa D., Diniz Lima E., Souza Paulino J.A., Dos Anjos Pontual M.L., Meira Bento P., Melo D.P. (2023). Age Determination on Panoramic Radiographs Using the Kvaal Method with the Aid of Artificial Intelligence. Dento Maxillo Facial Radiol..

[53] Dogan O.B., Boyacioglu H., Goksuluk D. (2024). Machine Learning Assessment of Dental Age Classification Based on Cone-Beam CT Images: A Different Approach. Dento Maxillo Facial Radiol..

[54] Granholm A., Alhazzani W., Møller M.H. (2019). Use of the GRADE Approach in Systematic Reviews and Guidelines. Br. J. Anaesth..

[55] Costa J., Montero J., Serrano S., Albaladejo A., López-Valverde A., Bica I. (2014). Accuracy in the Legal Age Estimation according to the Third Molars Mineralization among Mexicans and Columbians. Atención Primaria.

[56] Markovic E., Marinkovic N., Zelic K., Milovanovic P., Djuric M., Nedeljkovic N. (2021). Dental Age Estimation according to European Formula and Willems Method: Comparison between Children with and without Cleft Lip and Palate. Cleft Palate Craniofacial J..

[57] Shamim T. (2018). Forensic Pediatric Dentistry. J. Forensic Dent. Sci..

[58] Rath H., Rath R., Mahapatra S., Debta T. (2017). Assessment of Demirjian’s 8-Teeth Technique of Age Estimation and Indian-Specific Formulas in an East Indian Population: A Cross-Sectional Study. J. Forensic Dent. Sci..

[59] Jayaraman J., Wong H.M., King N., Roberts G. (2013). The French–Canadian Data Set of Demirjian for Dental Age Estimation: A Systematic Review and Meta-Analysis. J. Forensic Leg. Med..

[60] Lee S.-S., Kim D., Lee S., Lee U.-Y., Seo J.S., Ahn Y.W., Han S.-H. (2011). Validity of Demirjian’s and Modified Demirjian’s Methods in Age Estimation for Korean Juveniles and Adolescents. Forensic Sci. Int..

[61] Zaborowicz K., Biedziak B., Olszewska A., Zaborowicz M. (2021). Tooth and Bone Parameters in the Assessment of the Chronological Age of Children and Adolescents Using Neural Modelling Methods. Sensors.

[62] Murray P.E., Stanley H.R., Matthews J.B., Sloan A.J., Smith A.J. (2002). Age-Related Odontometric Changes of Human Teeth. Oral Surg. Oral Med. Oral Pathol. Oral Radiol. Endod..

[63] Verma M., Verma N., Sharma R., Sharma A. (2019). Dental Age Estimation Methods in Adult Dentitions: An Overview. J. Forensic Dent. Sci..

[64] Zheng Q., Ge Z., Du H., Li G. (2020). Age Estimation Based on 3D Pulp Chamber Segmentation of First Molars from Cone-Beam–Computed Tomography by Integrated Deep Learning and Level Set. Int. J. Leg. Med..

[65] Putra R.H., Doi C., Yoda N., Astuti E.R., Sasaki K. (2021). Current Applications and Development of Artificial Intelligence for Digital Dental Radiography. Dentomaxillofacial Radiol..

[66] Demirjian A., Goldstein H., Tanner J.M. (1973). A New System of Dental Age Assessment. Hum. Biol..

