# Supplementary material for: Performance of Artificial Intelligence Models Designed for Automated Estimation of Age Using Dento-Maxillofacial Radiographs—A Systematic Review
Source: Diagnostics (Basel). 2024 May 22;14(11):1079. doi: 10.3390/diagnostics14111079 (PMC11172066; doi:10.3390/diagnostics14111079)
Supplement: Supplementary file 1 [file diagnostics-14-01079-s001.zip › diagnostics-2977440-supplementary.pdf]

Supplementary Materials

Table S1: **Risk of Bias and Applicability Concerns**

		**RISK OF BIAS**				**APPLICABILITY CONCERNS**		
SI No	Authors	Patient Selection	Index Test	Reference Standard	Flow And Timing	Patient Selection	Index Test	Reference Standard
1.	Bunyarit SS et al. [28]	LOW	LOW	LOW	LOW	LOW	LOW	LOW
2.	Mualla N et al. [29]	LOW	LOW	LOW	LOW	LOW	LOW	LOW
3.	Galibourg A et al. [30]	LOW	LOW	LOW	LOW	LOW	LOW	LOW
4.	İsa AT al.[31]	LOW	LOW	LOW	LOW	LOW	LOW	LOW
5.	Wallraff S et al. [32]	LOW	LOW	LOW	LOW	LOW	LOW	LOW
6.	Kim S et al. [33]	LOW	LOW	LOW	LOW	LOW	LOW	LOW
7.	Shen S et al. [34]	LOW	LOW	LOW	LOW	LOW	LOW	LOW
8.	Milošević D et al. [35]	LOW	LOW	LOW	LOW	LOW	LOW	LOW
9.	Hann et al.36]	LOW	LOW	LOW	LOW	LOW	LOW	LOW
10.	Baydoğan MP et al.[37]	LOW	LOW	HIGH	LOW	LOW	LOW	HIGH
11.	Pintana P et al.[38]	LOW	LOW	LOW	LOW	LOW	LOW	LOW
12.	Saric et al. [39]	LOW	LOW	LOW	LOW	LOW	LOW	LOW
13.	Shen et al.[40]	LOW	LOW	LOW	LOW	LOW	LOW	LOW
14.	Wang et al.[41]	LOW	LOW	LOW	LOW	LOW	LOW	LOW
15.	Kumagai A et al.[42]	LOW	LOW	LOW	LOW	LOW	LOW	LOW
16.	Yeom HG et al.[43]	LOW	LOW	LOW	LOW	LOW	LOW	LOW
17.	Kahm SH et al.[44]	LOW	LOW	LOW	LOW	LOW	LOW	LOW
18.	Aljameel S et al.[47]	LOW	LOW	LOW	LOW	LOW	LOW	LOW
19.	Rin Kim et al.[46]	LOW	HIGH	HIGH	HIGH	LOW	HIGH	HIGH
20.	Murray J et al.[47]	LOW	LOW	LOW	LOW	LOW	LOW	LOW
21.	Zaborowicz M et al.[48]	LOW	LOW	LOW	LOW	LOW	LOW	LOW
22.	MU CC et al.[49]	LOW	LOW	LOW	LOW	LOW	LOW	LOW
23.	Wang J et al.[50]	LOW	LOW	LOW	LOW	LOW	LOW	LOW
24.	Sharifonnasabi F et al.[51]	LOW	LOW	LOW	LOW	LOW	LOW	LOW
25.	Pereira de Sousa et al.[52]	LOW	LOW	LOW	LOW	LOW	LOW	LOW
26.	Dogan B et al.[53]	LOW	LOW	LOW	LOW	LOW	LOW	LOW
